# Robust generalization of tuning to self-induced sensation

**DOI:** 10.1016/j.isci.2025.112563

**Published:** 2025-05-02

**Authors:** Rozana Ovsepian, David Souto, Alexander C. Schütz

**Affiliations:** 1 Sensorimotor Learning Unit, University of Marburg, 35032 Hessen, Germany; 2 School of Psychology and Vision Sciences, University of Leicester, Leicester LE1 7RH, UK; 3 Center for Mind, Brain and Behavior (CMBB), Marburg, Gießen & Darmstadt, 35032 Hessen, Germany

**Keywords:** Biological sciences, Clinical neuroscience, Natural sciences, neuroscience, Psychology, Social sciences

## Abstract

Perceptual and sensorimotor learning is often specific to the trained stimuli and movement parameters. This specificity also applies to recalibrating sensory and motor maps, such as saccadic eye movements in response to systematic visual errors. Here, we show that the perceptual recalibration of stationarity during smooth pursuit eye movements generalizes to untrained eye movement speeds. During smooth pursuit, the retinal image motion of the stationary surround (reafference) must be compensated to maintain perceptual stability. Prior research revealed that the predicted reafference signal is continuously updated through interactions between the motor command and experienced retinal motion and is specific to movement direction and visual field location. Here, we show that stationarity recalibration transfers across pursuit speeds. The generalization pattern reveals two distinct mechanisms: a multiplicative gain for decreasing predicted reafference signals and a constant shift for increasing signals. The former is consistent with a gain control model of smooth pursuit.

## Highlights

• Recalibration of sensory consequences in smooth pursuit generalizes across different speeds
• Two recalibration mechanisms are identified, depending on the background motion direction
• Gain-control when background moves in the same direction as pursuit
• Fixed shift when background moves in the opposite direction as pursuit

## Graphical abstract

**Figure:**
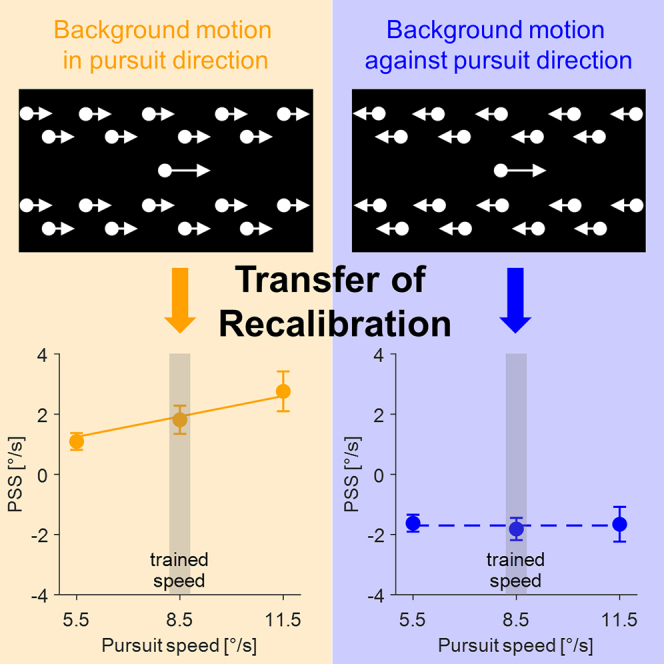

## Introduction

Smooth pursuit eye movements are used to track moving objects of interest. The movement of the eyes induces retinal motion signals, called reafference, which must be compensated to perceive the veridical head-centered motion of objects in the environment. This compensation is achieved by integrating information from two different sources: a purely retinal signal, encoding the velocity of the retinal image motion, and an eye movement signal that encodes the eye velocity.^1^^,^^2^^,^^3^^,^^4^^,^^5^^,^^6^^,^^7^^,^^8^ The eye movement signal is thought to be based on the efference copy or corollary discharge of the motor command, which is used to predict the sensory consequences of the eye movement, creating the *predicted reafference* (*reference signal*). The relative magnitude of the retinal velocity and the estimated eye movement velocity determines the perceived head-centric velocity.^9^

Any underestimation or overestimation of velocities by either signal can result in inaccurate motion perception. The Filehne illusion, where stationary objects appear to move in the direction opposite to a smooth pursuit eye movement,^10^^,^^11^ and the Aubert-Fleischl phenomenon, where a moving object seems to move more slowly when tracked with the eyes,^12^^,^^13^^,^^14^^,^^15^ are classic examples of signal misestimation. Both cases can be explained by assuming that the gain of the retinal signal is higher than the gain of eye movement signal.^3^^,^^16^ An alternative explanation assumes that the brain underestimates the speed of pursued objects, because of the uncertainty in sensory signals encoding motion. As a result, the brain relies more heavily on a Bayesian prior that assumes that objects in the world are stationary, causing the target to appear slower than it is.^17^ Previous research has shown that the prediction of the self-generated retinal motion (reference signal) is continuously recalibrated, based on a direction-selective interaction between the motor command of the pursuit, and the retinal flow experienced during the eye movement.^18^ As the eyes track a moving object across a coherently moving visual background, the brain gradually adjusts the reference signal to null the experienced motion of the background. Moreover, this calibration effect is specific to the exposed visual hemifield.^19^

These findings align with the characteristic specificity of learning in the human sensorimotor system, which is often closely tied to the stimuli and actions being trained. For example, perceptual learning, defined usually as an improvement in performance, is often highly stimulus specific.^20^ This means that improvements are typically confined to the specific features of the trained stimulus, such as its orientation or location, with little transfer to untrained stimuli. Similarly, habituation to sensory consequences of saccadic eye movements—the brain’s ability to predict and suppress visual disruptions caused by rapid eye movements—is highly dependent on the precise parameters of the saccades, such as their size, speed, or direction.^21^

In this study, we show that the recalibration of sensory consequences during smooth pursuit eye movements presents a different pattern: it generalizes robustly to untrained eye movement speeds. The pattern of generalization reveals two different types of recalibration: a multiplicative gain when recalibrating to a slower retinal flow and a constant shift when recalibrating to a faster retinal flow. The first mechanism is compatible with a gain control model of smooth pursuit.^22^ These findings reveal surprisingly flexible mechanisms underlying the brain’s ability to maintain perceptual stability—a key function for interacting effectively with the world.

## Results

**Figure 1:**
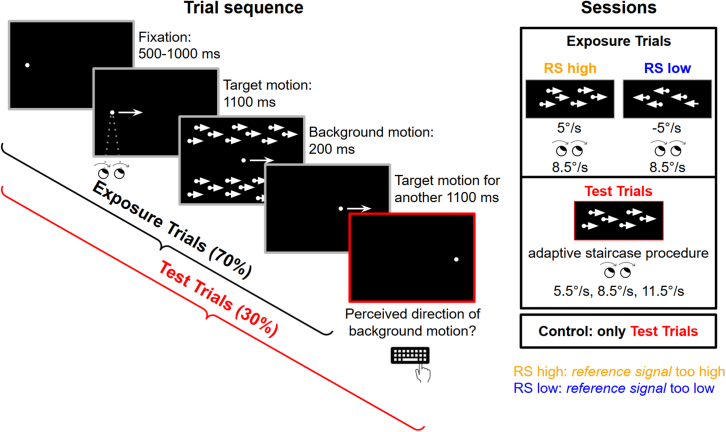
Trial structure and experimental sessions In all trials, observers pursued a dot that moved horizontally across the screen at a constant speed. Halfway through the dot’s path, a random dot background pattern was displayed for 200 ms. The background pattern moved coherently at a constant speed either to the left or to the right. In test trials, observers had to indicate the direction of the background motion. In exposure trials (70% of trials in exposure sessions), the pursuit target moved at 8.5 °/s and the perception of motion during pursuit was manipulated in two conditions. In the “reference signal too high” (RS high) condition, the background pattern moved at 5 °/s congruently with the direction of eye movements, simulating an overcompensated reafference signal. Conversely, in the “reference signal too low” (RS low) condition, the background pattern moved at −5 °/s, meaning it moved opposite to the direction of eye movements, simulating an undercompensated reafference signal. The resulting effects on which background motion is perceived as stationary (point of subjective stationarity, PSS) were tested at three pursuit speeds (5.5 °/s, 8.5 °/s, 11.5 °/s). The control condition was always completed first, and it only included test trials. Each observer completed all conditions for all pursuit speeds in a counterbalanced order, totaling 1,380 trials across 9 experimental sessions.

To investigate the recalibration of the reference signal during pursuit, 30 observers were asked to execute horizontal smooth pursuit while judging the direction of motion (horizontal optic flow) presented briefly in the background. In the middle of the pursuit target trajectory, a background pattern moving horizontally at 5 °/s was presented for 200 ms. When the background is static, the reafferent retinal image flow is opposite to the pursuit eye movement velocity. By moving the background in the direction of pursuit or opposite the direction of pursuit we simulate what would happen if the *reference signal* over- or underestimated the sensory consequences of a pursuit eye movement.^18^ In exposure trials (70%), the background moved either in the same direction as the eye movements (*reference* signal too high, RS high), reducing retinal motion velocity, and simulating an overcompensated reafference signal, or in the opposite direction (*reference* signal too low, RS low), enhancing retinal motion velocity and simulating an undercompensated reafference signal. In test trials (30%), observers were asked to report the perceived direction of the background motion, the velocity of which was varied to estimate the point of subjective stationarity (PSS). While in exposure trials, we trained observers only with a pursuit speed of 8.5 °/s, in test trials, we tested them at three pursuit speeds (5.5 °/s, 8.5 °/s, and 11.5 °/s) to assess generalization of recalibration (Figure 1).

### Recalibration effect

**Figure 2:**
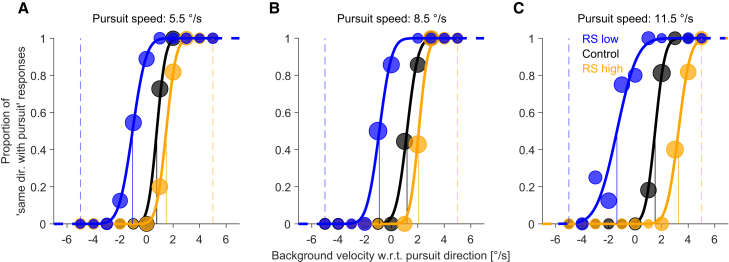
Recalibration of the reference signal in a representative observer (A–C) Example psychometric functions of one observer for the different conditions (color coded). The *x* axis represents background velocity with respect to (w.r.t.) the pursuit direction, with positive and negative values indicating motion in or against the direction of pursuit, respectively. The dashed lines indicate the background velocity in exposure trials (5 °/s in or against the direction of pursuit). Each dot represents binned proportion responses only for visualization. Each panel corresponds to a different pursuit speed (A: 5.5 °/s, B: 8.5 °/s, C: 11.5 °/s) during the test trials. Although we trained the observer in exposure trials only at a pursuit speed of 8.5 °/s, the reference signal—reflected by the PSS—shifted in the direction of motion during exposure in all tested pursuit speeds.

To determine if exposure to background motion during pursuit affected the perception of stationarity, we calculated psychometric functions from which the PSS can be derived, comparing control and exposure conditions (RS high, RS low). Figure 2 displays the results of a representative observer. Although we trained the observer in exposure trials only at a pursuit speed of 8.5 °/s, reference signal estimates—as reflected by the PSS—shifted in the direction of background motion during exposure in all tested pursuit speeds.

**Figure 3:**
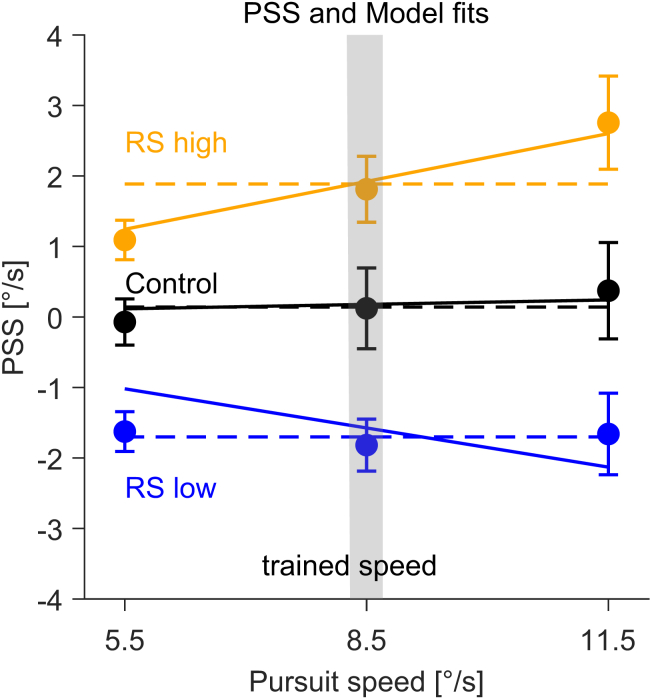
Group-level results (*n* = 30) PSS against different pursuit speeds for the different conditions (color coded). Data points represent the average across observers. Error bars represent 95% confidence intervals. Solid and dashed lines represent the fits of the gain and the shift model, respectively. The *y* axis represents background velocity with respect to the pursuit direction, with positive and negative values indicating motion in or against the direction of pursuit, respectively. The gray shaded area shows the pursuit speed at which observers were trained (8.5 °/s).

To quantify the recalibration, we analyzed the PSS across the different conditions for all tested pursuit speeds (Figure 3). A positive PSS indicates that the background had to move in the same direction as the pursuit target (leading to a slower retinal speed) to be perceived as stationary, whereas a negative PSS indicates that the background had to move in the opposite direction to the pursuit target (leading to a faster retinal speed) to appear stationary. The mean PSS in the control condition (no exposure trials) was −0.07 °/s (±0.88 STD), 0.12 °/s (±1.53), and 0.37 °/s (±1.82) for the pursuit speeds of 5.5 °/s, 8.5 °/s, and 11.5 °/s, respectively. Therefore, observers were overall well calibrated during the control condition, with mean PSSs close to 0°/s, consistently with previous findings.^23^^,^^24^ The mean PSS in the RS high condition were 1.09 °/s (±0.75), 1.81 °/s (±1.25), and 2.75 °/s (±1.77) and in the RS low condition were −1.62 °/s (±0.75), −1.81 °/s (±0.99), and −1.66 °/s (±1.55) for the corresponding pursuit speeds. Hence, the PSS was clearly different between the RS high and RS low conditions and shifted in the direction of motion presented during exposure trials.

To quantify the recalibration effect, we fitted our PSS data with two simple models: a gain model and a shift model. In the gain model, the slope (1 − gain) reflects the fraction of the pursuit speed that needs to be subtracted from the perceived motion of the background to make it appear stationary:

(Equation 1) PSS=(1−gain)∗pursuitspeed

**Figure 4:**
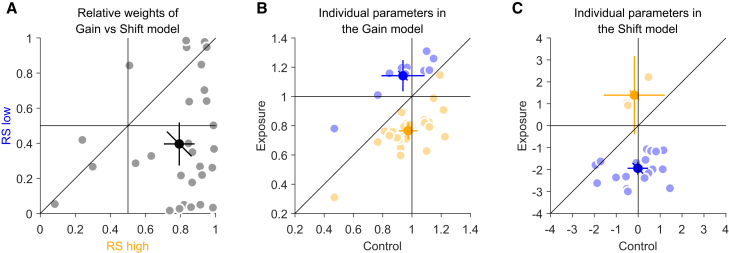
Individual-level results (A) Relative weights of the gain versus the shift model in the RS high and RS low conditions. Gray data points represent individual observers; the black data point the average across observers. Error bars represent 95% confidence intervals. (B) Individual parameters from the gain model in the control, RS high, and RS low conditions. The darker orange data point represents the mean gain value across observers whose data were best fit by the gain model in the RS high condition, while the darker blue data point represents the mean gain value across observers whose data were best fit by the gain model in the RS low condition. Lighter-colored data points represent individual observers in each of the two conditions (RS high, orange; RS low, blue). Error bars represent 95% confidence intervals. (C) Individual parameters from the Shift model for all observers in the control, RS high, and RS low conditions. The darker orange data point represents the mean shift value across observers whose data were best fit by the Shift model in the RS high condition, while the darker blue data point represents the mean shift value across observers whose data were best fit by the shift model in the RS low condition. Lighter-colored data points represent individual observers in each of the two conditions (RS high, orange; RS low, blue). Error bars represent 95% confidence intervals.

The gain indicates the extent to which the visual system compensates for the eye movements, 1 indicating perfect compensation and 0 no compensation. This means the PSS is expected to increase or decrease proportionally with pursuit speed. In contrast, the shift model assumes a constant shift of the PSS across all pursuit speeds (Figure 3). First, we fitted the models to the average data across observers. Remarkably, model comparison showed that the gain model best fitted the averaged data from the RS high condition (model weight of 0.99), while the shift model best fitted the averaged data from the RS low condition (model weight of 0.99). Second, we fitted the models to the data of individual observers and calculated the relative weights of each model in the RS high and RS low conditions based on their Bayesian information criterion (BIC) values and derived model weights with 0.5 indicating no model better fitting the data. The average model weight in the RS high condition was with 0.79 (±0.23) significantly greater than 0.5 (t(29) = 6.85, *p* = 1.595x10^−7^), providing conclusive evidence for a gain modulation in the RS high condition. Although in the RS low condition, the average model weight was with 0.39 (±0.33) not significantly different from 0.5 (t(29) = −1.73, *p* = 0.094), the mean weights were significantly different from each other in the RS high and the RS low conditions (t(29) = 5.77, *p* = 2.9836x10^−6^). This suggests that the models differed in how well they could explain the data in the two conditions (Figure 4A).

Moreover, we investigated the individual parameters from the gain model in the control, RS high and RS low conditions. The gain model was the best fit for 27 of 30 observers in the RS high condition and for 10 of 30 observers in the RS low condition. The mean gain value across observers whose data were best fit by the gain model in the RS high condition was 0.76 (±0.14), while the mean gain value across observers whose data were best fit by the gain model in the RS low condition was 1.14 (±0.15). The mean gain value in the control condition for all observers was 0.98 (±0.15), indicating nearly perfect compensation. The mean gain value from the RS high condition was significantly lower than one (t(26) = −8.45, *p* **=** 6.2 × 10^−9^) and significantly lower than the value in the control condition (t(26) = −9.404, *p* **=** 8.0 × 10^−10^) which indicates that the exposure to background motion in the direction of pursuit decreased the compensation of the reference signal proportionally to the pursuit speed. In contrast, the mean gain value from the RS low condition was significantly greater than one (t(9) = 3.02, *p* = 0.015) and significantly greater than the value in the control condition (t(9) = 7.252, *p* **=** 4.806 × 10^−5^), indicating that observers, after being exposed to background motion against the pursuit direction, overcompensated for their self-induced retinal motion (Figure 4B).

Similarly, we plotted the individual parameters from the shift model for all observers in the control, RS high and RS low conditions. The shift model was the best fit for 20 of 30 observers in the RS low condition and for 3 of 30 observers in the RS high condition. The mean shift value across observers whose data were best fit by the shift model in the RS high condition was 1.39 (±0.71), while the mean shift value across observers whose data were best fit by the Shift model in the RS low condition was −1.94 (±0.63). The mean shift value in the control condition for all observers was 0.14 (±1.28). The mean shift value from the RS low condition was significantly lower than zero (t(19) = −13.86, *p* = 2.188 × 10^−11^) and significantly lower than the value in the control condition (t(19) = −8.413, *p* = 7.88 × 10^−8^), which indicates that observers perceived the background as stationary when it was moving at 1.94 °/s opposite to the pursuit, irrespectively of pursuit speed (Figure 4C).

### Pursuit gain

**Figure 5:**
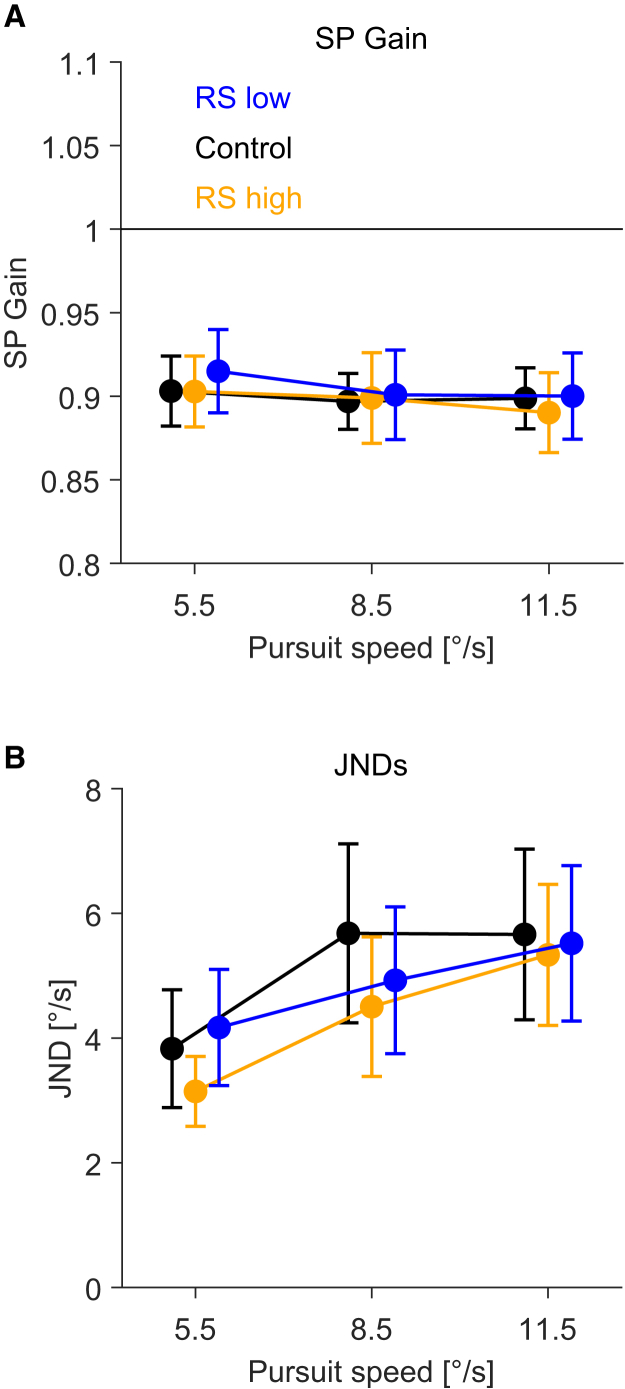
Smooth pursuit gain and JNDs (A) Pursuit gain (eye velocity/target velocity) during the background stimulus presentation. The filled circles indicate the mean values for each condition and pursuit target speed across observers. Data points are horizontally jittered to improve visibility. Error bars represent 95% confidence intervals. The horizontal dashed line represents the ideal pursuit gain (gain = 1). (B) Just-noticeable difference (JND) against pursuit speeds for the different conditions (color coded). The filled circles indicate the mean values for each condition across observers. Data points are horizontally jittered to improve visibility. Error bars represent 95% confidence intervals.

To rule out that the recalibration effect (Figures 2 and 3) was caused or affected by variations in eye movements, we calculated pursuit gain (eye velocity/target velocity) during the presentation of the background stimulus (Figure 5A). We found that pursuit gain was not significantly different between conditions (F(2, 58) = 0.75, *p* = 0.477), which is consistent with previous findings.^19^ There was also no significant effect of pursuit speed (F(2, 58) = 1.83, *p* = 0.169), and the differences in pursuit gain were in the order of 0.01, leading to negligible differences in retinal speeds of stationary patterns between 0.055 and 0.115 °/s. Recalibration effects were more than one order of magnitude larger than these differences in retinal speeds.

### Discriminability

Finally, we investigated if motion direction discriminability was affected by pursuit speed. If discriminability depends on the head-centered speed of background motion, it should be fairly independent of pursuit speed. The mean just-noticeable-difference (JND) in the control condition was 3.82 (±2.53), 5.68 (±3.84), and 5.66 (±3.66) for pursuit speeds of 5.5 °/s, 8.5 °/s, and 11.5 °/s, respectively. In the RS high condition, it was 3.14 (±1.50), 4.50 (±2.30), and 5.33 (±3.03), and in the RS low condition, it was 4.17 (±2.50), 4.92 (±3.15), and 5.51 (±3.34). The trend of increasing JNDs with higher pursuit speeds means that discriminability in motion direction deteriorated when pursuit speed increased. A repeated measures ANOVA revealed that there was a significant main effect of pursuit speed (F(2,58) = 15.79, *p* = 3.35 × 10^−^^6^), but no significant difference between the three conditions (F(2,58) = 2.41, *p* = 0.098) and no significant interaction between condition and pursuit speed (F(4,116) = 0.67, *p* = 0.612). It is important to note that the maximal difference in JNDs between conditions was quite small, about 2 °/s, compared to the maximal difference in PSSs of about 4 °/s, and also compared to the differences in pursuit speeds of 6 °/s between the slowest and the fastest speed.

## Discussion

This study reveals that the recalibration of sensory consequences (i.e., the perception of self-induced motion during smooth pursuit) can generalize to eye movement speeds not previously exposed to. Taken together with previous studies, this highlights that the recalibration effect in smooth pursuit can be specific in terms of pursuit direction^18^ and location within the visual field^19^ and broad—in terms of pursuit speed—at the same time. Furthermore, the generalization across pursuit speeds differs from the habituation to sensory consequences of saccadic eye movements, which does not generalize across saccade amplitudes.^21^ This would fit with the notion that while saccades with different amplitudes are programmed separately, there is a single mechanism controlling pursuit at different speeds.^22^

The prediction of sensory consequences of smooth pursuit most likely involves several brain areas. Neurons in the middle temporal area (MT) signal information about the background’s speed and direction on the retina. Neurons in the medial superior temporal area (MST) integrate this retinal motion with an efference copy of the motor command to signal object motion irrespective of pursuit.^25^^,^^26^ The efference copy might originate in the frontal eye fields (FEF), where neurons show a gain modulation during pursuit.^22^ The different patterns of recalibration in our results suggest that this network can react in different ways to discrepancies between predicted and experienced sensory consequences of pursuit.

### Generalization across speeds

The generalization of recalibration across pursuit speeds but not across pursuit direction or stimulus location might be based on neural tuning properties and reflect ecological demands.

The broad speed tuning of neurons in MT and MST regions^25^^,^^27^^,^^28^ might enable the brain to recalibrate the reference signal across a range of different speeds, rather than being specific to a single pursuit speed. The gain-control of smooth pursuit eye movements^22^ also supports this idea. Whereas, the direction-selectivity in MT and MST with separate neuronal populations encoding motion signals for specific directions^29^^,^^30^^,^^31^ might explain why the recalibration of sensory predictions in one direction may not transfer to other directions.^18^ Similarly, MT neurons have small receptive fields^32^^,^^33^^,^^34^^,^^35^^,^^36^ and are highly location-specific. Thus, the observed spatially localized recalibration effect could originate in MT or in the transmission of signals from MT to MST.^19^ Taken together, it seems that the brain encodes direction and location in a way that optimizes perceptual accuracy for specific tasks.

While it seems that smooth pursuit is governed by a single mechanism that can operate across speeds, the saccadic system has a different, more discrete profile; despite the broad tuning for saccade amplitude and direction in superior colliculus (SC),^37^ the habituation to the sensory consequences for saccades does not generalize well across amplitudes, speeds, or directions.^21^

This specificity likely reflects the functional demands of the saccadic system, where accuracy in discrete gaze shifts is crucial. Generalization in that case could lead to interference between movements with different amplitudes or direction, reducing precision. In contrast, the continuous nature of smooth pursuit allows for broader generalization across speeds.

### Different generalization patterns

The different patterns of generalization across pursuit speeds revealed that exposure to background motion in or against pursuit invoked different mechanisms of recalibration. In the RS high condition, where the background pattern moved in the same direction as the eye movement, the retinal motion signal was reduced, making the background appear to move slower, simulating an overcompensated predicted reafference signal. In response, the brain recalibrated its reference signal, proportionally to the actual pursuit speed. This recalibration took the form of a gain control mechanism, where the response of the system was based on the input (in this case, pursuit speed). This proportional adjustment is compatible with a genuine recalibration of the relationship between pursuit and its sensory consequences,^18^ where the brain fine-tunes its internal model to better match the dynamic relationship between eye movements and sensory input. In contrast, in the RS low condition, where the background pattern moved opposite to the direction of the eye movement, the retinal motion signal was increased, making the background motion more pronounced, and simulating an undercompensated predicted reafference signal. To recalibrate, the system shifted the reference signal by a constant amount across all pursuit speeds, aligning the perceived background motion with the expected motion. This underestimation introduced a constant bias that remained the same regardless of the pursuit speed, suggesting that the brain predicts a fixed external motion in the scene and applies a constant adjustment to correct for the bias.

The asymmetry in smooth pursuit recalibration mechanisms fits into a broader set of perceptual asymmetry patterns in sensory and motor processing. For example, motion sensitivity is greater for motion toward the fovea (centripetal) than for motion directed away from it (centrifugal), while sensitivity to motion in depth is greater in the lower visual field than in the upper visual field.^38^ Similarly, recalibration in vestibulo-ocular adaptation is context dependent, adjusting to the observer’s environmental needs.^39^ Additionally, the adaptation of saccade amplitudes is accomplished by different mechanisms for shortening and lengthening of amplitudes and these mechanisms also differ in the generalization across contexts (for a review, see Pélisson et al.^40^). These phenomena often reflect how the brain prioritizes or processes information differently depending on context, direction, or other environmental factors.

Anisotropies in the way the visual system deals with reafferent motion signals have been uncovered in different contexts. With fixational eye movements (drifts), it was found that objects that move in the same direction as retinal slip (reafferent motion) are found to be more stable (they are perceived as slower) than elements moving in the same direction as the eyes, at similar amounts of retinal slip.^41^^,^^42^ This finding is consistent with the attenuation of expected signals, where sensation that is consistent with the expected consequences of eye movements would be attenuated. Consistently, it has been shown that temporal contrast sensitivity^43^ and motion smear^44^ are selectively reduced for motion opposite to pursuit. The integration of global motion is too suppressed in the opposite direction.^45^

### Further implications

We also found that the discriminability of motion direction degraded with increasing pursuit speeds. Since the calculation of head-centered speed (and direction) requires combining retinal speed and eye speed, either retinal motion signals or the efference copy signal might become less reliable at higher pursuit speed. For instance, faster eye movements create greater retinal slips, and signal-dependent noise might lead to poorer discriminability. Also, faster retinal motion signals may fall outside the optimal speed range for MT neurons,^28^ further reducing discriminability. Similarly, the efference copy might be less reliable for larger signals, i.e., faster pursuit speeds.

Patients with schizophrenia frequently exhibit abnormal smooth pursuit eye movements, characterized by reduced pursuit gain (the ratio of eye velocity to target velocity), increased variability in tracking accuracy, and impaired prediction and adjustment for sensory outcomes of self-generated actions.^46^^,^^47^ These abnormalities suggest that there are disruptions in the integration of motor commands (efference copy) and sensory feedback—processes critical for compensating for self-induced sensory changes. Deficits in the gain mechanism, which prevent proportional recalibration of the reference signal to pursuit speed, may underlie their inability to effectively cancel out self-induced motion.^48^

To summarize, we showed that the recalibration of sensory consequences of smooth pursuit eye movements generalizes to eye movement speeds that were not previously exposed. We identified two distinct recalibration mechanisms: a gain-control mechanism, which enables recalibration to scale proportionally with pursuit speed when the eyes move in the direction of the background motion, and a fixed recalibration mechanism, which results in a consistent PSS regardless of pursuit speed when the eyes move opposite to the background motion direction.

### Limitations of the study

The recalibration of sensory consequences of pursuit transferred from a pursuit speed of 8.5 °/s to speeds of 5.5 °/s and 11.5 °/s. While this spans the range of pursuit speeds typically investigated,^49^^,^^50^^,^^51^ pursuit can reach up to speeds of 40 °/s. Therefore, we cannot exclude that the transfer might be reduced or even eliminated completely for larger differences in speed. However, neurophysiological studies showed that the tuning for speeds in area MT and especially area MST is quite broad^25^^,^^27^^,^^28^ which could be the neural basis for the generalization across speeds. In this case, the transfer could extend to speeds beyond the tested range.

Although there was a considerable variability in the PSS values in the baseline measurements (control condition), the mean PSS value for all tested speeds was close to zero (Figure 3). According to the Filehne illusion,^10^ a static background is seen as moving opposite to the direction of the pursuit.^17^ Therefore, the PSS during the control condition should have been positive—the background needs to be shifted in the direction of the movement for it to be perceived as stationary. The lack of the Filehne illusion is consistent with some previous findings,^23^^,^^24^ and could arise from a combination of factors. First, with the repeated exposure to background motion, observers may have adapted during the experiment, learning to better compensate for their eye movements. Second, the contrast of the background dots was very low, given the requirement of excluding external references provided by the screen edges by juxtaposing neutral density filters. This could lead to underestimating the speed of retinal motion. Since head-centric angular velocity is the addition of retinal velocity and pursuit velocity^52^ and assuming that low-contrasts do not lead to a reduction in pursuit velocity estimates, a reduced retinal signal would lead to a lower Filehne illusion, as noted before.^17^ Third, even though the motion direction of the pursuit target was randomized (right/left), the motion was still predictable (moving horizontally), which could have improved the ability to track accurately, leading to a reduced illusion. Indeed, the pursuit gain was high across all tested speeds (Figure 5B).

Since we interleaved exposure and test trials, our experimental paradigm assessed the immediate consequences of retinal motion during pursuit on perceived stationarity. It would be interesting to present the test trials after a delay following the exposure trials (no interspersed exposure and test trials). This would allow to observe if the system maintains the adjustment over time or whether it quickly reverts back to baseline. If the system is just adjusting in real-time to each exposure trial, the observed effects might reflect an ongoing adaptation rather than true recalibration, which involves a more long-term change in sensory processing. Along the same lines, it would be useful to evaluate the strength of recalibration while manipulating the duration of the background presentation.

## Resource availability

### Lead contact

Further information and requests for resources should be directed to and will be fulfilled by the first author, Rozana Ovsepian (ovsepian@staff.uni-marburg.de).

### Materials availability

This study did not generate new specimens or materials. All images are included in the text.

### Data and code availability

• Eye-movement data and perceptual data have been deposited at https://doi.org/10.5281/zenodo.15078450 and are publicly available as of the date of publication. DOIs are listed in the key resources table.
• This paper does not report original code.
• Any additional information required to reanalyze the data reported in this paper is available from the lead contact upon request.

## Acknowledgments

This work was supported by the Research Cluster “The Adaptive Mind,” funded by the Excellence Program of the Hessian Ministry of Higher Education, Science, Research and Art.

## Author contributions

Conceptualization, R.O., D.S., and A.C.S.; methodology, R.O. and A.C.S.; investigation, R.O.; writing – original draft, R.O.; writing – review and editing, R.O., D.S., and A.C.S.; funding acquisition, A.C.S.; resources, R.O. and A.C.S.; visualization, R.O. and A.C.S.

## Declaration of interests

The authors declare no competing interests.

## STAR★Methods

### Key resources table

**Table:** 

REAGENT or RESOURCE	SOURCE	IDENTIFIER
**Deposited data**		

Psychophysical data	This study	Database: https://doi.org/10.5281/zenodo.15078450

**Software and algorithms**		

MATLAB	The MathWorks	https://www.mathworks.com/products/matlab.html, RRID:SCR_001622
Psychtoolbox	Psychtoolbox	http://psychtoolbox.org/, RRID:SCR_002881

**Other**		

Eyelink 1000+ eye tracker	SR Research	https://www.sr-research.com/products/eyelink-1000-plus/, RRID:SCR_009602
VIEPixx monitor	VPixx Technologies	https://vpixx.com/products/viewpixx/, RRID:SCR_013271
Optical Filters	LEE Filters	https://leefilters.com/
Arduino board	Arduino board mega 2560 Rev3	https://store.arduino.cc/en-gr/products/arduino-mega-2560-rev3

### Experimental model and study participant details

We measured data from 30 observers (13 males, 17 females, 18–44 years) with normal or corrected-to-normal vision. Except for the first author, all observers were compensated with 8€ per hour and were naive with respect to the experiment’s purpose. Before taking part, observers provided written informed consent. Ethical approval for the experiment was obtained from the local ethics committee of the Department of Psychology at the University of Marburg (proposal 2017-27k).

### Method details

#### Apparatus

Observers were seated in front of a VIEWPixx monitor (VPixx Technologies Inc, Quebec, Canada) of 51.5 × 29 cm size, a refresh rate of 120 Hz and a resolution of 1920 × 1080 pixels, positioned 60 cm away, in a darkened room. The observer’s head was stabilized by a head-and-chin rest. Eye movements were recorded with EyeLink 1000 Plus system (SR Research Ltd., Ontario, Canada) at 1000 Hz. Stimuli were programmed by using the Psychophysics toolbox^53^^,^^54^^,^^55^ in MATLAB (R2017a) (Mathworks, Natick, US), and the EyeLink Toolbox.^56^

To minimize external visual references that could be used to estimate motion, we reduced the amount of light emitted by the screen by placing neutral density filters in front of the screen. The resulting luminance of white and black pixels was 0.16 cd/m^2^ and 0.0002 cd/m^2^ (nominal value, below the measurement threshold of a UDT Instruments Optometer 370), respectively. To prevent dark adaptation, we programmatically turned-on 10 LED lamps for 3 s (using an Arduino board) every twenty trials. Data analysis was conducted with MATLAB (R2021a).

#### Stimuli

The fixation target was a white cross with a diameter of 0.8°. The pursuit target was a white dot with a diameter of 0.4°. The random dot background pattern had a density of 0.44 dots/deg^2^, and consisted of white dots with a diameter of 0.2°. The background pattern was presented on the whole screen, except for a horizontal gap of 10° height at the center of the screen, where the pursuit target traveled horizontally.

#### Procedure

We tested how recalibration of self-induced retinal motion (predicted reafference, or reference signal) during pursuit transfers from one pursuit speed (8.5 °/s) to three pursuit speeds (5.5, 8.5 and 11.5 °/s). In exposure trials, the background moved at 5 °/s either in the same direction as the pursuit (RS high) or in the opposite direction (RS low). The different pursuit speeds and the different exposure conditions were tested in separate sessions. In each exposure condition, 140 exposure trials were randomly interleaved with 60 test trials to measure the point of subjective stationarity (PSS), totaling 200 trials. Control conditions only contained test trials, to measure the calibration without any exposure. Observers were in general quite well calibrated in the Control conditions before the exposure. Each observer completed all conditions for all pursuit speeds in a counterbalanced order, totaling approximately 5 h and 30 min of recording across nine experimental sessions.

In each trial, observers fixated on a white cross positioned 6.6°, 10.2°, or 13.8°, depending on the pursuit speed (5.5 °/s, 8.5 °/s, or 11.5 °/s) either on the left or right of the screen center. After pressing the spacebar to start the trial, the fixation cross was replaced by the pursuit target, which remained stationary for 500–1000 ms before it started moving horizontally at a constant speed. Midway through the trajectory, 1100 ms after the motion onset, a background cloud of random dots, moving coherently at a constant velocity either left or right, was displayed for 200 ms. The pursuit target continued moving until it reached an equivalent eccentricity on the opposite side.

In test trials—as well as in the baseline trials (Control condition)—observers were required to indicate the perceived motion direction of the background by pressing the left or the right button on the keyboard. The background speed was controlled by an adaptive staircase procedure, starting at a speed of 5 °/s to the left or right and then was varied in 1 °/s steps to estimate the point of subjective stationarity (PSS). After each trial, the background speed was adjusted based on the observer’s previous response: if they indicated that the background motion was in the pursuit direction, the speed of the dots in the next trial was decreased; if they indicated that the background was moving opposite to the pursuit direction, the speed was increased. This procedure effectively enabled the identification of the PSS. In exposure trials, observers simply followed the pursuit target—which was always moving at 8.5 °/s—while experiencing background motion at a fixed velocity of either +5 °/s or −5 °/s within a session.

### Quantification and statistical analysis

Psychophysical and eye-position data were stored for further offline analysis. Eye velocity was obtained by differentiation of the eye-position data. We identified saccades by using the EyeLink algorithm (velocity threshold = 22 °/s, acceleration threshold = 3,800 °/s^2^). Trials in which blinks or saccades occurred during the presentation of the background motion were discarded from the eye movement analysis and from the psychophysical analysis, which amounted to 15.7% of trials on average per individual (2.03–35.5%, SD = 9.66%). To calculate the pursuit gain, we only used the eye movement data during a time window when the background stimulus was presented.

We fitted our PSS data with two different linear models. We used PSS as the dependent variable and the different pursuit target speeds as the predictors. For each model, we calculated the Bayesian information criterion (BIC) across the averaged data, and the difference between the individual BIC values and the lowest value. The BIC takes into account the residual sum of squares (RSS), the number of free parameters *k*, and the number of observations *n.*^57^ Finally, we calculated the relative weights for each model,^58^ to estimate which one fits best our data.

(Equation 2) $$\text{BIC}=\text{n}\ln\left(\text{RSS}/n\right)+\text{k}\ln\left(n\right)$$

(Equation 3) $$\Delta \text{BI}C_{i}=\text{BI}C_{i}-\text{BI}C_{\min}$$

(Equation 4) $$\text{pi}=\frac{e^{-0.5\Delta ΒIC_{i}}}{\sum_{r=1}^{R}e^{-0.5\Delta ΒΙC_{r}}}$$

The models were fit to the average data across observers, as well as to the data of individual observers.

To derive psychometric functions, the responses of observers were transformed into proportions indicating whether the background moved in the same direction as the pursuit target. PSS was identified as the background velocity yielding, on average, an equal number of leftward and rightward responses. Cumulative Gaussian psychometric functions were fitted using Psignifit 2.5.6. in Matlab.^59^ The standard deviation of the cumulative Gaussian was used to estimate the JND values.
